# Fluid biomarkers in familial frontotemporal dementia: progress and prospects

**DOI:** 10.3389/fneur.2025.1663609

**Published:** 2025-08-18

**Authors:** Mengyao Guo, Linyuan Qin, Hanlin Cai, Ruihan Wang, Caimei Luo, Feng Yang, Shiyu Feng, Hui Gao, Qin Chen

**Affiliations:** Department of Neurology, West China Hospital of Sichuan University, Chengdu, China

**Keywords:** fluid biomarker, familial frontotemporal dementia, MAPT, GRN, *C9orf72*

## Abstract

Familial frontotemporal dementia (FTD) is a genetically heterogeneous disease with various clinical manifestations, making it difficult to diagnose. There are three main gene mutations in familial FTD: repeat expansion in chromosome 9 open reading frame 72 (*C9orf72*), microtubule-associated protein tau (*MAPT*), and progranulin (*GRN*). These mutations can produce corresponding changes in fluid biomarkers years before symptoms appear. Therefore, biomarkers play a vital role in the diagnosis and treatment of familial FTD. In this review, we highlight fluid biomarkers in the blood and cerebrospinal fluid (CSF) that contribute to the clinical diagnosis of familial FTD, the study of disease pathophysiological mechanisms, and possibly be used as outcome endpoints in future clinical trials.

## 1 Introduction

Frontotemporal dementia (FTD) is a common form of early-onset dementia that predominantly impacts the frontal and temporal lobes, exhibiting diverse clinical and pathological characteristics (1, 2). It is characterized by significant personality, behavioral changes, and cognitive impairment (3). Approximately 10–30% of FTD are hereditary, exhibiting a distinct autosomal dominant inheritance pattern (4). An autosomal dominant inheritance pattern has been reported in 10–25% of families with FTD (5, 6). For clarity, we define familial FTD as cases with an identifiable autosomal dominant mutation. In contrast, sporadic FTD refers to phenotypically similar but genetically unconfirmed cases without a clear family history. The most common genetic mutations that cause familial FTD include repeat expansions in chromosome 9 open reading frame 72 (*C9orf72*), microtubule-associated protein tau (*MAPT*), and progranulin (*GRN*) (7). Less common genetic reasons include mutations in *TBK1*, *TARDBP*, *VCP*, *FUS*, *CHMP2B*, *SQSTM1*, and *UBQLN2* (8). The pathological proteins generated by different genes display considerable heterogeneity, and the clinical manifestations arising from identical gene mutations and harmful protein deposits vary significantly depending on their deposition locations, posing a considerable challenge for clinical diagnosis (4, 9).

*C9orf72* is the most common genetic cause of familial FTD (10). FTD associated with *C9orf72* was caused by the amplification of the GGGGCC hexanucleotide repeat in the non-coding region of the gene. The length of this pathogenic repeat sequence may vary from 30 to several 1,000, whereas healthy individuals often possess fewer than 30 repetitions (11). The repeat sequences in *C9orf72* can be transcribed into abnormal RNA transcripts. The RNA transcripts can be subsequently translated into dipeptide repeat proteins (DPRs), such as poly (GA), poly(GR), poly(PR), poly(PA), and poly(GP), which have toxic effects on neurons (12). *C9orf72*-associated FTD is predominantly linked to TDP-43 type B pathology, characterized by cytoplasmic inclusions of TDP-43 protein in neurons and glial cells, especially in the frontal and temporal lobes (13).

The gene encoding the progranulin (*GRN*) was also found on chromosome 17 (14). This mutation produces an aberrantly shortened progranulin mRNA transcript, resulting in diminished quantities of full-length functional progranulin proteins. The haploinsufficiency of progranulin disrupts normal lysosomal and neuronal functioning, thereby contributing to the pathophysiology of FTD-TDP (15).

The *MAPT* gene is located on chromosome 17q21.3, contains 16 exons, spans approximately 150 kb, encodes tau protein, and significantly influences neuronal integrity. Mutations in *MAPT* result in abnormal aggregation of tau proteins, ultimately leading to degeneration of glutamatergic neurons (16). The most prevalent *MAPT* mutations were P301L, V337M, R406W, and N27. The P301 mutation decreases tau affinity for microtubules while enhancing its aggregation and phosphorylation, thereby increasing the pathological accumulation of tau protein and resulting in neurodegeneration (17, 18).

Although the clinical manifestations and pathological characteristics can be linked, their association is typically limited (19). Therefore, sensitive biomarkers for familial FTD are necessary due to the heterogeneity of the disorder. Fluid biomarkers are molecules or chemicals present in physiological fluids that can fluctuate and indicate the presence of a disease (Table 1).

**Table 1:** Clinical phenotypes and differential diagnoses of familial FTD subtypes.

Genetic mutation	Chromosomal localization	Main clinical phenotypes	Common differential diagnoses
*MAPT*	17q21.1	bvFTD, PPA, Parkinsonism	PSP, AD, DLB
*GRN*	17q.21.32	bvFTD, nfvPPA	Corticobasal syndrome, stroke-related aphasia
*C9orf72*	9p21.2	bvFTD, nfvPPA, svPPA,	Amyotrophic lateral sclerosis, schizophrenia, bipolar disorder

MAPT, microtubule-associated protein tau gene; GRN, progranulin gene; C9orf72, hexanucleotide expansion in chromosome 9; bv-FTD, behavioral variant frontotemporal dementia; PPA, primary progressive aphasia; PSP, Progressive supranuclear palsy; AD, Alzheimer’s disease; DLB, dementia with Lewy bodies; nfv-PPA, nonfluent variant primary progressive aphasia; sv-PPA, semantic variant primary progressive aphasia; ALS, amyotrophic lateral sclerosis; CBS, corticobasal syndrom.

Research has shown that biomarkers in the blood and cerebrospinal fluid (CSF) have potential for investigating FTD. However, sporadic FTD accounts for more than 70% of all clinical cases, and its complicated etiology includes environmental factors, somatic mutations, genetic mosaicism, and challenging biomarker research (20). In contrast, familial FTD offers a more genetically defined framework, facilitating mechanistic investigation and reliable biomarker confirmation. Additionally, developing findings indicate that over 60% of sporadic FTD individuals exhibit overlapping endosomal-lysosomal biomarker profiles with familial subtypes, suggesting shared downstream pathogenic pathways (21). Thus, the familial FTD biomarker framework also serves as a potential reference for the classification, stratification, and therapeutic targeting of sporadic FTD (22).

Biomarkers can be classified into several functional categories. Diagnostic biomarkers help confirm the presence of disease, while predictive biomarkers identify individuals at risk of developing symptoms. Prognostic biomarkers provide information on disease progression, and monitoring biomarkers track treatment response or disease severity over time. Different biomarkers may serve distinct roles depending on genetic subtype, disease stage, and clinical presentation.

Overall, identifying fluid biomarkers of familial FTD is crucial for tracking disease development, anticipating treatment outcomes, and investigating potential pathophysiological alterations associated with the condition. In this review, we describe the most recent developments in fluid biomarkers associated with familial FTD.

## 2 Methods

We conducted an electronic search of the MEDLINE, PubMed, and Embase databases using a combination of several keywords. The following search terms were used as keywords to identify all relevant studies: (“FTD” OR “FTD” OR “frontotemporal dementia” OR “lobar degeneration” OR “frontotemporal lobar degeneration”) AND (“microtubule associated protein tau” OR “*MAPT*” OR “Progranulin” OR “*GRN*” OR “Progranulin” OR “*GRN*”) AND (“biomarker”). Related studies that contained these keywords from the references were also searched for potentially qualified studies. Studies were excluded if they were (1) reviews without original data; (2) unrelated to familial FTD; or (3) not written in English. We also excluded studies that did not differentiate between genetic subtypes of FTD.

## 3 Discussion

### 3.1 Biomarkers in the blood

Current research on familial FTD in the blood mainly involves three environments: serum, plasma, and small extracellular vesicles (sEVs). We reviewed and summarized these biomarkers in the blood (Table 2).

**Table 2:** Biomarkers in the blood.

No.	References	No. of subjects	Measurement	Biosamples	Biomarker	Main findings
1	Dols-Icardo et al. (29)	7 C9 vs. 62 NC	Elisa	Plasma	Progranulin	Progranulin levels in C9 carriers did not differ from those in patients who did not carry the amplification mutation.
2	Carecchio et al. (92)	1 *GRN*	Elisa	Plasma	Progranulin	Progranulin levels are lower in carriers of *GRN* mutations.
3	Sleegers et al. (28)	9 *GRN* vs. 9 NC vs. 22 HC	Elisa	Plasma	Progranulin	Progranulin levels were reduced in both affected and unaffected null mutation carriers compared with NC, and allowed perfect discrimination between carriers and noncarriers
4	Meeter et al. (30)	7 *GRN* vs. 28 PS *GRN* vs. 29 NC	Elisa	Plasma	Progranulin	*GRN* mutation carriers had lower plasma progranulin levels than controls, without any overlap between the groups.
5	Galimberti et al. (27)	19 *GRN* vs. 64 PS vs. 77 NC	Elisa	Plasma	Progranulin	Progranulin levels in patients and asymptomatic carriers were significantly decreased compared with NC.
6	Sellami et al. (32)	129 *GRN* vs. 31 PS *GRN* vs. 133 HC	Elisa	Plasma	Progranulin	Progranulin expression in plasma predicts *GRN* mutation status, independently of symptom onset proximity,but is not predictive of phenotype or age at onset.
7	Benussi et al. (31)	79 *GRN* vs. 50 NC	Elisa	Plasma	Progranulin	In mutation carriers, progranulin levels were already reduced at more than 30 years before expected symptom onset compared with NC.
8	Ghidoni R et al. (26)	309 HC vs. 72 *GRN* null mutation carriers vs. 3 *GRN* missense mutation carriers	Elisa	Plasma	Progranulin	Plasma progranulin protein cutoff level of 61.55 ng/mL that identifies, with a specificity of 99.6% and a sensitivity of 95.8%, null mutation carriers among subjects attending to a memory clinic.
9	Panman et al. (93)	35*GRN* vs. 56 PS *GRN* vs. 35 HC	Simoa	Serum	Nfl	Nfl as an early biomarker for disease onset in FTD-*GRN*
10	Saracino et al. (37)	165 HC vs. (54 C9 + 48 *GRN*) vs. (48 PS C9 + 37 PS *GRN*)	Simoa	Plasma	Nfl	*GRN* patients had higher levels than C9 and greater progression rates.
11	Meeter et al. (36)	71 HC vs. 62 PS (34 *GRN* vs. 14 C9 vs. 14 *MAPT*) vs. 101 patients (53 *GRN* vs. 29 C9 vs. 19 *MAPT*)	Elisa	Serum	Nfl	higher levels in patients than in PS and HC without a difference between the latter two groups. *GRN* patients had higher Serum Nfl levels than *MAPT* patients, both did not differ from C9 patients. Serum Nfl did not differ between the three presymptomatic groups.
12	Rohrer et al. (94)	28HC vs. 74 FTD (9 C9 vs11 *MAPT* vs. 4 *GRN*)	Simoa	Serum	Nfl	Concentrations were significantly higher than HC in both the C9 and *MAPT* subgroups with a trend to a higher level in the *GRN* subgroup.
13	Van Der Ende et al. (35)	59 patient (25 *GRN* vs. 24 vs. C9 vs. 10 *MAPT*) vs. 149 PS (79 PS *GRN* vs. 46 PS C9 vs. 24 PS *MAPT*) vs. 127 NC	Simoa	Plasma	Nfl	Baseline Nfl was elevated in symptomatic carriers compared with PS and NC.
14	Wilke et al. (39)	117 C9 vs. 104 *GRN* vs. 49 *MAPT* vs. 174 NC	Simoa	Serum	Nfl pNfH	Nfl increase preceded the hypothetical clinical onset by 15 years and concurred with brain atrophy onset, whereas pNfH increase started close to clinical onset.
15	Silva-Spínola et al. (65)	(20 *GRN* vs. 13 C9 vs. 30 sporadic-FTD) vs. 37 AD vs. 37 HC	Elisa	Serum	Nfl	FTD patients had significantly higher serum Nfl levels than both AD patients and HCs
16	Linnemann et al. (24)	66 PS (22 C9 vs. 29 *GRN* vs. 15*MAPT*) vs. 4 converter (3C9 vs. 1*GRN*) vs. patient (7C9 vs. 8*GRN* vs. 9 *MAPT*) vs. 60 NC	Simoa	Serum	Nfl	Nfl revealed an excellent consistency and high reliability Serum and plasma Nfl were largely comparable.
17	Heller et al. (45)	114 C9 carriers (74PS vs. 40C9) vs. 119 *GRN* carriers (88PS vs. 31*GRN*) vs. 53 *MAPT* carriers (34PS vs. 19 *MAPT*) vs. 183 NC	Simoa	Plasma	(1) GFAP (2) Nfl	(1) Plasma GFAP concentration was significantly increased in symptomatic *GRN* mutation carriers, but not in those with C9 expansions, *MAPT* mutations or the presymptomatic groups. (2) GFAP concentration was significantly positively correlated with age both in controls and in the majority of the disease groups, as well as with Nfl concentration.
18	Katisko et al. (43)	26 C9 vs. 31 *GRN* vs. 3 *MAPT* vs. 105 HC vs. 170 NC	Simoa	Serum	total TDP-43	Total levels of TDP-43 in the serum are decreased especially in FTD patients with the C9 repeat expansion.
19	Suárez-Calvet et al. (41)	10 C9 vs. 5*GRN* vs. 51 NC vs22 HC	Elisa	Plasma	(1) total TDP-43 (2) pTDP-43	(1) Subjects carrying a C9 repeat expansion or *GRN* mutations had significantly increased levels of plasma pTDP-43 compared with subjects with FTD without a mutation and with HC. (2) Total TDP-43 levels were slightly decreased in the C9 and the *GRN* groups compared with the FTD group, Subjects with *GRN* mutations also showed decreased levels of plasma total TDP-43 levels compared with controls. (3) Plasma pTDP-43 levels correlated inversely with plasma total TDP-43 levels in the entire group
20	Bellini et al. (55)	40 sporadic FTD vs. 33 C9 vs. 45 *GRN* vs. 43 HC	Elisa	Plasma sEVs	Cathepsin D	A progressive reduction in plasma cathepsin D moving from the intermediate to *C9orf72* pathological expansion carriers. The diagnostic performance of t plasma small extracellular vesicles (sEVs) was fairly high in *GRN*/*C9orf72* and Sporadic FTD.
21	Heikkinen et al. (56)	82 HC vs. 89 NC vs. (21 C9 vs. 31 *GRN* vs. 3 *MAPT*)	Simoa	Serum	Cathepsin S	There was no difference in serum cathepsin S levels between *GRN* and HC or NC. Comparing C9 HRE-carrying FTD patients to HC or to C9 HRE-non-carrying FTD patients did not reveal any statistically significant differences in the serum cathepsin S levels.
22	Van Der Ende et al. (58)	74NC vs. 104 PS (46*GRN* vs. 42 C9 vs. 16 *MAPT*) vs. patient (11 *GRN* vs. 28 C9 vs. 7 *MAPT*)	Elisa	Serum	C2, C3	The elevated complement protein levels in plasma remained statistically significant only in C9.
23	Esteras et al. (57)	7 *GRN* vs. 33HC vs. 8MCI vs. 35 AD vs. 4DLB vs. 20 PD vs. 10ALS vs. 5 PSP	MS	Peripheral blood mononuclear cells	CaM	CaM levels were not increased in the other neurodegenerative disorders.

HC, healthy control individuals, no sign of neurological disease; NC, non-carriers; PS, presymptomatic carriers; FTD, frontotemporal dementia; *GRN*, FTD with progranulin; *MAPT*, FTD with microtubule-associated protein tau; *C9orf72*, FTD with the chromosome 9 open reading frame 72 repeat expansion; MCI, patients with a diagnosis of mild cognitive impairment; AD, patients with probable Alzheimer’s disease; DLB, patients diagnosed with dementia with Lewy bodies; PD, patients with probable Parkinson’s disease; ALS, patients diagnosed with amyotrophic lateral sclerosis; PSP, patients diagnosed with progressive supranuclear palsy; Nfl, neurofibrillary light chain; pNfH, neurofibrillary high chain; GFAP, glial fibrillary acidic protein; TDP-43, TAR DNA-binding protein 43; pTDP-43, phosphorylated TAR DNA-binding protein 43; small extracellular vesicles, sEVs.

#### 3.1.1 Progranulin

Progranulin serves as a significant biomarker for detecting *GRN* mutations in FTD (23). Many studies have shown that a considerable reduction in progranulin levels is typical of individuals with *GRN* mutations and is not linked to other forms of familial FTD (14, 15, 24–27). Progranulin levels assessed by enzyme-linked immunosorbent assay (ELISA) accurately distinguished between *GRN* mutation carriers and healthy individuals, demonstrating a specificity of 99.6% and a sensitivity of 95.8% (28). Dols-Icardo et al. further demonstrated that progranulin levels remained unaffected by the *C9orf72* mutation, indicating that progranulin serves as a specific biomarker for *GRN*-related FTD (29). Meeter et al. found that progranulin levels in the blood of a considerable cohort of presymptomatic *GRN* mutation carriers were significantly lower than those of age-matched healthy controls. This suggests that progranulin can be employed not only in symptomatic individuals but also in identifying mutation carriers before symptom manifestation, thus functioning as an effective early diagnostic tool (30). Benussi et al. also found that progranulin, as a “status” biomarker for *GRN* mutations, may serve as an early warning indicator prior to symptom manifestation, although exhibits minimal correlation with the rate of illness development (31). Sellami et al. suggested that this detection method is economically viable and appropriate for screening, allowing for patient selection without the need for costly genetic testing (32). In summary, measurement of progranulin in the blood can identify carriers of *GRN* mutations and can be used as an early diagnosis and cost-effective screening method. However, the presence of progranulin indicates only a possible gene mutation and has no correlation with the degree of neurodegeneration in the brain.

#### 3.1.2 Neurofilament light chain protein

The neurofilament light chain (Nfl) is a subunit of neurofilaments (Nfs), which are cylindrical proteins located in the cytoplasm of neurons (33). Nfl is found in dendrites, neuronal bodies, and axons, contributing to the structural stability of neurons (34). Although Nfl is not a disease-specific biomarker, its elevated levels have been consistently observed in a range of neurodegenerative disorders, including FTD. Van Der Ende et al. found that serum Nfl levels were significantly elevated in symptomatic carriers relative to presymptomatic carriers and non-carriers, indicating clinical progression and highlights the potential value of serum Nfl as a candidate selection tool for disease progression (35). In *GRN*-associated FTD, serum Nfl levels are elevated two–three times 2–4 years prior to the onset of clinical symptoms, enabling dynamic monitoring of neuronal axonal damage and disease progression through regular blood tests (36). Saracino et al. further discovered that the concentration of Nfl in individuals with the *GRN* mutation was markedly elevated compared to those with the symptomatic *C9orf72* mutation, indicating that Nfl may represent varying rates of pathological disease progression (37). Moreover, while Nfl levels are elevated in *MAPT* mutation carriers compared to healthy controls, the magnitude of elevation is typically lower than that observed in *GRN* and *C9orf72* carriers, which is consistent with the relatively slower disease progression associated with *MAPT*-related pathology (38). Wilke et al. indicated that Nfl levels progressively increased over the 15 years preceding symptom manifestation, implying its potential as an early prognostic marker for familial FTD development (39). Linnemann et al. observed that the robust constancy of Nfl across multicenter investigations renders it a suitable biomarker for clinical trials, particularly for assessing disease progression and treatment response (24). Overall, Nfl levels in the blood indicate the underlying clinical burden of familial FTD and demonstrate the potential for differentiating genetic subtypes, particularly with high sensitivity and predictive value in *GRN* mutation carriers. Its levels progressively elevate years prior to the onset of symptoms and thus could be used to predict early diagnosis.

#### 3.1.3 TAR DNA binding protein 43

TAR DNA-binding protein 43 (TDP-43) is an RNA-binding protein that induces aberrant protein aggregation in the cytoplasm during pathological conditions (40). The TDP-43 protein assay typically has two forms: (1) total TDP-43 levels and (2) phosphorylated TDP-43 (pTDP-43), which exhibit distinct alterations across several genetic subtypes of FTD. Suarez-Calvet et al. found that pTDP-43 levels increased in *C9orf72* and *GRN* mutation carriers, whereas total TDP-43 levels decreased, indicating an inverse relationship between pTDP-43 and total TDP-43 (41). Changes in TDP-43 levels may indicate abnormalities in protein metabolism and disease mechanisms, particularly in processes related to protein aggregation and neurodegeneration. Under pathological conditions, TDP-43 undergoes post-translational modifications, primarily phosphorylation, resulting in cytoplasmic mislocalization and aggregation. Unlike total TDP-43, which includes both functional and diseased forms, pTDP-43 is disease-specific and serves as a hallmark of TDP-43 proteinopathies (42). Katisko et al. further observed that total TDP-43 levels were markedly diminished in individuals with the *C9orf72* mutation, indicating that TDP-43 may possess diagnostic significance in *C9orf72*-associated FTD (43). Thus, TDP-43 and its phosphorylated form may function as biomarkers for the future diagnosis of familial FTD, especially in individuals carrying *C9orf72* and *GRN* mutations.

#### 3.1.4 Glial fibrillary acidic protein

Glial fibrillary acidic protein (GFAP) is considered a marker of astrocyte activation and may significantly contribute to the pathophysiology of *GRN*-associated FTD (44–46). GRN mutations result in diminished quantities of functional progranulin protein, thereby compromising lysosomal function and exacerbating neuro-inflammation. This failure in astrocytes induces a reactive state marked by increased cytokine release and modified homeostatic support, thereby facilitating disease development (47).

Activated astrocytes release inflammatory mediators that compromise neuronal connections and disrupt lysosomal function, hence exacerbating neurodegeneration (48, 49). Heller et al. showed that GFAP levels are significantly elevated in *GRN* mutations, particularly prior to symptom onset, implying that GFAP could serve as a valuable early biomarker for identifying the risk in these patients (45). More importantly, GFAP levels were found to be positively correlated with neurofilament light chain (Nfl) levels, which indicates the potential for employing both as dynamic surveillance indicators, which could improve the accuracy of disease classification (50) To summary, the *GRN* mutation is strongly associated with an intensified inflammatory response, and the increase in GFAP may indicate pathogenic activation of astrocytes in the early stages of the disease. Consequently, GFAP may serve as a possible biomarker for the early detection of *GRN*-associated FTD, and could elucidate the neuroinflammatory mechanisms underlying the disease. Nevertheless, existing research on GFAP expression in other genetic subgroups of FTD is limited. This gap highlights the need for further research.

#### 3.1.5 Lysosomal proteases

Recent investigations on lysosomal proteins in familial FTD have predominantly concentrated on cathepsin D and S. Cathepsin D, an aspartic protease found in lysosomes that participates in proteolytic metabolism and regulates the digestion of hormones and antigens. It has been shown to be correlated with neurodegenerative alterations (51, 52). Animal models carrying *GRN* and *C9orf72* mutations have shown a marked reduction in plasma Cathepsin D activity, suggesting a role in disease pathogenesis (53, 54). Consistent with these findings, human studies have reported a notable decrease in Cathepsin D levels in individuals with *GRN* and *C9orf72* mutations along with a progressive decline in cathepsin D plasma levels from *C9orf72* intermediate expansion carriers to *C9orf72* pathological expansion carriers. Pathogenic expansions are characterized by hexanucleotide repeats exceeding 30 G4C2 repetitions, whereas intermediate expansions are defined as consisting of 12–30 hexanucleotide repeats. This suggests a dose-dependent influence of *C9orf72* expansion on cathepsin D plasma levels (55). The decrease in Cathepsin D did not occur after the onset of symptoms but was already apparent during the asymptomatic phase. Thus, cathepsin D may serve as a presymptomatic biomarker. Furthermore, the diagnostic efficacy of cathepsin D concentration in extracellular vesicles as a criterion for distinguishing FTD patients from healthy controls was notably high (AUC = 0.85), exhibiting a sensitivity of 75.4% and a specificity of 76.7%. This indicates that extracellular vesicle-associated Cathepsin D may serve as a diagnostic biomarker, especially for identifying patients with pathogenic *GRN* or *C9orf72* mutations.

Although Cathepsin D is an aspartic protease localized within lysosomes, Cathepsin S is a cysteine protease that is predominantly expressed by microglia and is involved in antigen presentation and immune regulation. In contrast to Cathepsin D, Heikkinen et al. found that serum Cathepsin S levels were not significantly different between patients with familial FTD and healthy controls. No significant differences were observed among FTD subtypes, including GRN and C9orf72 mutant carriers, MAPT mutation carriers, or sporadic cases, indicating that Cathepsin S is not a reliable biomarker for differentiating clinical, genetic, or pathological groupings (56).

Overall, Cathepsin D showed a notable decrease in *GRN* and *C9orf72* carriers associated with disease progression and elevated copy number in *C9orf72*, thus signifying mutant gene carriers and pathological conditions, thereby functioning as a potential biomarker for screening and early detection of familial FTD. Further investigation of additional lysosomal protein types may uncover novel biologically significant biomarkers of familial FTD.

#### 3.1.6 Other biomarkers in the blood

Calmodulin (CaM) in peripheral cells used as a potential biomarker to investigate the association between familial FTD and other degenerative disorders. Elevated CaM levels have been specifically observed in peripheral blood mononuclear cells in patients with AD but not in those with other neurodegenerative disorders. Consequently, CaM could aid in differential diagnosis in the differential diagnosis of familial FTD, providing complementary value to established core AD biomarkers, such as phosphorylated tau species, MTBR-tau isoforms, and Aβ42/40 ratio (57).

The complement system plays an important role in neuroinflammation and synaptic clearance and its activation may facilitate neurodegeneration. Van Der Ende et al. identified markedly increased concentrations of the complement proteins C2 and C3 in the plasma of *C9orf72* mutant carriers, indicating a potential association between complement system overactivation and illness development (58). As these alterations manifest before symptom onset, testing for C2 and C3 may facilitate the early identification of illness risk in carriers of the *C9orf72* mutation. Increased levels of complement proteins in various neurological illnesses suggest a generalized overexpression of the complement system rather than gene-or disease-specific upregulation (59–61). Thus, although complement proteins may assist in identifying a higher disease risk in *C9orf72* carriers, their diagnostic specificity across other FTD genotypes remains unclear. Future research comparing complement levels in genetically related subtypes of FTD may further clarify potential gene-specific effects.

### 3.2 Biomarkers in the CSF

CSF biomarkers can accurately indicate disease progression with minimal interference. We reviewed and summarized the CSF biomarkers of familial FTD (Table 3).

**Table 3:** Biomarkers in the CSF.

No.	Reference	No. of subjects	Measurement	Biosamples	Biomarker	Findings
1	Meeter et al. (30)	7 *GRN* vs. 16 PS *GRN* vs. 12 NC	Elisa	CSF	Progranulin	CSF progranulin in carriers was 39% of that in NC, without overlap of the levels between the groups.
2	Morenas-Rodríguez et al. (64)	74 HC vs. 90 MCI vs. 73 AD vs. 32FTD (non-*GRN*) vs. 11(PSP + CBS) vs. 23 DLB	Elisa	CSF	Progranulin	Polymorphism rs5848 in *GRN* iNfluenced CSF progranulin levels, but APOEɛ4 allele did not.
3	Meeter et al. (36)	71 HC vs. 62 PS (34*GRN* vs. 14C9 vs. 14 *MAPT*) vs. 101 patients (53 *GRN* vs. 29 C9 vs. 19 *MAPT*)	Elisa	CSF	Nfl	CSF Nfl levels in patients were more than eight times higher than in PS and HC, without a difference between the latter two groups. *GRN* patients had higher CSF Nfl levels than C9 and *MAPT* patients.
4	Silva-Spínola et al. (65)	20 *GRN* vs. 13 C9 vs. 30 sporadic-FTD	Elisa	CSF	Nfl	FTD patients had significantly higher CSF levels than both AD patients, NC and HC.
5	Carecchio et al. (66)	145 AD vs. 120 FTD (non-*GRN*) vs. 20 *GRN* vs. 38 HC	Elisa	CSF	Aβ_42_ total tau p-tau-181	*GRN* mutation carriers and HC did not differ significantly for any biomarker, whereas *GRN* negative FTD patients had higher tau levels than controls and *GRN* Thr272fs mutation.
6	Sato et al. (70)	80 AD vs. 74 4R tauopathy vs. 5 *MAPT* R406W vs. 98 HC	MS	CSF	p-tau_217_/t-tau_217_ × Aβ _42/40_	individuals with increased CSF pT217/T217 and normal Aβ 42/40 ratio, most of whom were *MAPT* R406W mutation carriers.
7	Kapaki et al. (67)	3 C9 vs. 2 *GRN* vs. 1VCP	Elisa	CSF	TDP-43 TDP-43 × pT/pT_181_	Genetic FTD is characterized by increased CSF TDP-43 and increased TDP-43 × pT/pT_181_ combination.
8	Woollacott et al. (76)	17HC vs. 64FTD (3 *GRN* vs. 4 *MAPT* vs. 3 C9)	Elisa,	CSF	sTREM2	CSF sTREM2 levels did not differ between FTD and HC or between clinical subgroups. However, *GRN* mutation carriers had higher levels than HC and *MAPT* or C9 mutation carriers.
9	E. L. van der Ende et al. (77)	(35 *GRN* +34 PS *GRN*) vs. (32 C9 + 6 PS C9) vs. 67 NC	Elisa	CSF	sTREM2	No group differences in sTREM2 levels were observed, and high levels were seen in a subset of *GRN*, but not C9, mutation carriers.
10	Woollacott et al. (78)	62 NC vs. 121 carriers (49 C9 vs. 49 *GRN* vs. 23 *MAPT*)	Elisa	CSF	sTREM2 YKL-40 chitotriosidase	Only chitotriosidase in *GRN* had a concentration significantly higher than controls. No group had higher sTREM2 or YKL-40 concentrations than NC.
11	Horie et al. (72)	88 HC vs. 28 bvFTD vs. 16 PSP vs. 15CBS vs. 80 AD vs. 8 *MAPT*	Immunoassay	CSF	MTBR-tau275, MTBR-tau282	Their study demonstrated that the MTBR-tau275/t-tau and MTBR-tau282/t-tau ratios were reduced in *MAPT*
12	Borrego-Écija et al. (79)	18 HC vs. 115 FTD (6 *MAPT* vs. 5 PS *MAPT* vs. 13 *GRN* vs. 13 C9)	Elisa	CSF	Gal-3	A significant elevation of Gal-3 levels in *MAPT* carrier samples compared to *GRN* carriers, C9, and HC samples. No statistically significant differences were found between *GRN* and C9 groups.
13	Schneider et al. (73)	(22 *GRN* vs. 11C9 vs. 5 *MAPT*) vs. 11NC	qPCR	CSF	miR-204-5p and miR-632	A significantly lower expression of miR-204-5p and miR-632 in symptomatic compared with PS in the genetic FTD cohort.
14	Van Der Ende et al. (80)	54 patient (15 *GRN* vs. 31 C9 vs. 8 *MAPT*) vs. 106 PS (47 PS *GRN* vs. 42 PS C9 vs. 17 *MAPT*) vs. 70 NC	Elisa	CSF	NPTX2	Symptomatic mutation carriers had lower NPTX2 concentrations than PS and NC.
15	Van Der Ende et al. (58)	74 NC vs. 104 PS (46 *GRN* vs. 42 C9 vs. 16 *MAPT*) vs. patient (11 *GRN* vs. 28 C9 vs. 7 *MAPT*)	Elisa	CSF	C1q, C3b	The elevated complement protein levels in CSF remained statistically significant only in C9 mutation carriers
16	Huang et al. (81)	3-month old *GRN*+/+ wild type (*n* = 4) and *GRN*−/− knock out (*n* = 4) mice and 19-month old *GRN*+/+ wild type (*n* = 4) and *GRN*−/− knock out (*n* = 4) mice human brain samples (21 *GRN* vs. 23 con) human CSF samples (13 *GRN* vs. 13 C9 vs. 12 *MAPT* vs. 14 cognitively normal controls)	Elisa	CSF	GPNMB	GPNMB levels were significantly increased in the CSF of FTD-*GRN* patients, but not in *MAPT* or C9 carriers.

HC, healthy control individuals, no sign of neurological disease; NC, non-carriers; PS, presymptomatic carriers; FTD, frontotemporal dementia; GRN, FTD with progranulin; *MAPT*, FTD with microtubule-associated protein tau; *C9orf72*, FTD with the chromosome 9 open reading frame 72 repeat expansion; bvFTD: behavioral variant frontotemporal dementia; MCI, patients with a diagnosis of mild cognitive impairment; AD, patients with a diagnosis of probable Alzheimer’s disease; DLB, patients diagnosed for dementia with Lewy bodies; PD, patients with probable Parkinson’s disease; ALS, patients with a diagnosis of amyotrophic lateral sclerosis; PSP, patients diagnosed of progressive supranuclear palsy; total tau, T; pT, the phosphorylated tau; amyloid β 42 (Aβ42); miR, MicroRNA; sTREM2, soluble TREM2; NPTX2, neuropentagramin 2; glycoprotein NMB, GPNMB.

#### 3.2.1 Progranulin

Progranulin has been examined in the CSF less extensively than in the blood, and the correlation between CSF and blood levels is relatively weak (62, 63). Unlike the blood test, which has a clear cut-off value, the evaluation of progranulin in CSF studies is uncertain. Research conducted by Meeter et al. indicated that individuals with *GRN* mutations exhibited markedly diminished progranulin levels, even prior to the onset of symptoms (30). Morenas-Rodríguez et al. found that CSF progranulin levels were strictly regulated and possessed no diagnostic utility except in cases of primary neurodegenerative dementia associated with *GRN* mutations (64). Thus, progranulin in CSF may serve as a specific biomarker for *GRN* mutations. However, precise cut-off value thresholds for potential clinical use necessitate further clarification.

#### 3.2.2 Neurofilament light chain (Nfl)

Consistent with findings in blood, CSF Nfl levels also exhibit a progressive increase during the presymptomatic phase and correlate with brain atrophy and clinical decline (65). Importantly, CSF and serum Nfl levels are strongly correlated (*r* = 0.87, *p* < 0.001), supporting the use of less invasive serum testing for longitudinal monitoring. Unlike blood-based measurements, CSF Nfl may offer slightly higher sensitivity in distinguishing symptomatic from presymptomatic carriers, particularly in early-stage *MAPT* or *GRN* associated cases (36). Nfl studies in both blood and CSF have shown similar results, suggesting that although non-specific, Nfl could be used as a predictive biomarker of disease progression.

#### 3.2.3 Amyloid and tau-related biomarkers

Amyloid beta (Aβ) and tau proteins are characteristic markers of Alzheimer’s (66, 67). Aβ PET imaging showed a low positivity rate in individuals diagnosed with FTD, particularly in cohorts with autopsy-confirmed diagnoses. Reimand et al. found that only 11.1% of patients with FTD showed Aβ PET positivity, primarily linked to concurrent AD rather than isolated FTD pathology (68). This supports the use of AD-associated biomarkers, including Aβ-PET or CSF Aβ42, as exclusionary tools in the context of FTD. Elevated levels of total tau (t-tau) and phosphorylated tau (p-tau), along with decreased Aβ42 in patients with AD, could be used as biomarkers to differentiate AD from FTD (69). The ratio of p-tau217/t-tau217 to Aβ 42/40 in CSF demonstrated efficacy as a composite biomarker to identify tau lesions in carriers of the *MAPT* R406W mutation, and could effectively distinguish them from cognitively normal individuals and those with other tauopathies (70). TDP-43 × p-tau/p-tau18 has also demonstrated diagnostic significance in familial FTD, particularly in association with the pathology of TDP-43. Kapaki et al. found that CSF TDP-43 levels, especially when analyzed alongside tau-based ratios (TDP-43 × t-tau/p-tau), were increased in genetic FTD cases with *GRN* and *C9orf72* mutations, highlighting their diagnostic relevance for TDP-43-driven subtypes (67). The microtubule-binding region (MTBR) of tau represents the central area of tau aggregates within the brain along with truncated C-terminal tau fragments found in the CSF (71). Horie et al. demonstrated that the MTBR-tau275/t-tau and MTBR-tau282/t-tau ratios were significantly reduced in MAPT-associated FTD cases compared with cognitively normal controls and patients with AD (72). This reduction likely reflects distinct aggregation patterns and epitope accessibility of tau filaments in primary tauopathies versus AD, underscoring the diagnostic specificity of MTBR-based tau measurements. Given the substantial overlap in biomarker expression across neurodegenerative diseases and the genotypic heterogeneity inherent in FTD, diagnosis relies more on multimodal biomarker panels and personalized interpretations than on a single biomarker.

#### 3.2.4 MicroRNA

MicroRNAs (miRNAs) within exosomes possess diagnostic potential for genetic FTD, and are gaining interest. Schneider et al. revealed a considerable reduction in the expression of miR-204-5p and miR-632 in symptomatic FTD, particularly among *carriers of GRN mutations* (73). In *GRN*-related FTD, downregulation of miR-204-5p and miR-632 leads to overexpression of the pro-apoptotic target HRK gene, which contributes to neuronal death in the frontal and temporal lobes (74, 75). However, because of the unique pathogenic mechanisms of different genotypes (such as *C9orf72* and *MAPT*), the expression of the aforementioned miRNAs did not show significant changes; thus, detection efficacy was limited. These findings suggest that miR may be an early detection method for the diagnosis of familial FTD; however, further evidence is needed.

#### 3.2.5 Soluble TREM2

Soluble TREM2 (sTREM2) serves as a biomarker for neuroinflammation and microglial activation. Woollacott et al. reported elevated sTREM2 levels in the CSF of symptomatic *GRN* mutation carriers, implying that sTREM2 may serve as a biomarker for neuronal damage in familial frontotemporal dementia (76). Conversely, Van der Ende et al. demonstrated no significant disparities in CSF sTREM2 levels between presymptomatic and symptomatic carriers of *GRN* or *C9orf72* mutations and noncarriers (77). Owing to the limited and contradictory evidence, it is currently difficult to determine the potential importance of this biomarker.

#### 3.2.6 Other biomarkers in the CSF

It was found that levels of chitotriosidase and Galectin-3 (Gal-3) were elevated in *MAPT* mutation carriers, potentially serving as markers of neuroinflammation and glial cell activation (78). Borrego-Écija et al. further showed that Gal-3 is significantly upregulated in *MAPT*-associated FTD, indicating its potential as a subtype-specific biomarker linked to neuroinflammation and glial cell activation (79). Van Der Ende et al. observed elevated levels of complement proteins in both CSF and plasma of genetic FTD cases through the GENFI study, further supporting the hypothesis that immune dysregulation is significant in disease pathology. These findings indicated that these proteins could function as biomarkers for *MAPT*-associated FTD subtypes. The observed heterogeneity among the various genetic forms of FTD, as noted by Van Der Ende et al., highlights the need for additional research to clarify the specific functions of these proteins in disease onset and progression.

Another potential biomarker is the decreased level of neuropentagramin 2 (NPTX2) in individuals with familial FTD, suggesting that NPTX2 may serve as a novel synaptic-derived biomarker of disease progression (80).

In animal experiments, glycoprotein non-metastatic melanoma protein B (GPNMB) levels were significantly elevated in the cerebrospinal fluid of FTD-*GRN* mice, whereas no such increase was observed in *MAPT* or *C9orf72* mice. GPNMB may serve as a specific biomarker for *GRN-*associated FTD, facilitating monitoring of disease onset, progression, and response to treatment. Additionally, GPNMB expression was increased in brain tissue from human *GRN*-associated FTD samples, consistent with the results from *GRN*-deficient mouse models (81).

In *C9orf72*-associated FTD, five dipeptide repeats (DPR) are generated by this gene. However, only poly (GP) levels are quantifiable and may serve as diagnostic markers for patient screening, such as blood tests (82, 83). Poly (GP) levels may be used for early disease detection, stratification of mutation carriers, and evaluation of therapeutic efficacy in clinical trials.

Despite these promising findings, most candidate biomarkers remain at an exploratory stage. Extensive longitudinal and multicenter studies across diverse populations are required to confirm their diagnostic, prognostic, and therapeutic monitoring utility in familial FTD.

### 3.3 Other biomarkers

In addition to the blood and CSF, there is a paucity of investigations on other fluid biomarkers for familial FTD. In a single longitudinal cohort investigation, salivary lactoferrin, which serves as a biomarker to differentiate AD from FTD, exhibited over 87% sensitivity and 91% specificity. Saliva is readily accessible, and sampling is straightforward and noninvasive. However, the quality of a specimen is affected by various factors that may hinder its development (84).

### 3.4 Measurement technique

Tremendous breakthroughs have been made in the assessment of fluid biomarkers of familial FTD. The predominant markers under investigation are proteins, and the three most frequently employed methods are ELISA, single-molecule array (Simoa), and mass spectrometry (MS). Elisa, while widely used, exhibits notable limitations. Its sensitivity is constrained, particularly in detecting markers during the early stages of the disease or at low concentrations, which may remain undetected due to insufficient levels. Additionally, its dynamic range is restricted, potentially limiting its ability to capture subtle changes in biomarker expression (85–87). The Simoa technique, which is widely utilized today, is an ultrasensitive method for detecting protein biomarkers through single-molecule counting. The detection limit of this technology can achieve levels on the order of FML (fg/mL) (88). Simoa demonstrates the capability to identify extremely low concentrations of neural markers in comparison to Elisa, which is especially significant for the early diagnosis of familial FTD. Simoa instruments are costly, kits are relatively expensive, and their application scope is somewhat restricted. MS can be used to identify and measure biomarkers linked to familial frontotemporal dementia through analysis of proteins, metabolites, and peptides (89). This method is appropriate for the concurrent detection of multiple related biomarkers in familial FTD, identifying markers present in very low quantities, and facilitating the observation of minor changes in the early stages of the disease (90). However, MS imposes stringent criteria for sample extraction and pretreatment, resulting in a relatively complex operational process (91). Quantitative polymerase chain reaction (qPCR) has emerged as a complementary tool, particularly for the detection of nucleic acid–based biomarkers. The new techniques improve the sensitivity and specificity of the test, but they also require stricter procedures for processing specimens, which must be carried out in a licensed laboratory to ensure the accuracy of the test and its potential use in clinical diagnosis (Table 4).

**Table 4:** Technique in familial FTD.

Technique	Application in familial FTD	Advantage	Limitation
Elisa	To detect relevant protein markers	Cheap and easy to use	Different specifications, and the detection accuracy is weak
Simoa	Ultrasensitive detection of low concentrations of FTD markers in blood or cerebrospinal fluid	High sensitivity for early detection of low protein concentrations	Expensive, requiring specialized equipment, and not yet widely used in the clinic
MS	To identify FTD-associated protein modifications and mutations and identify potential biomarkers	To analyze unknown molecules, detect modified proteins, suitable for proteomics	Expensive and sample preparation is complicated
qPCR	To quantitatively analyze FTD related gene expression changes	To analyze DNA and RNA	Primer design is required, and RNA analysis requires additional reverse transcription steps. Not suitable for protein

Elisa, assay; Simoa, single molecule array; MS, Mass spectrometry; qPCR, quantitative PCR.

### 3.5 Challenges and limitations

Despite an increasing number of studies on fluid biomarkers of familial FTD, significant challenges and limitations remain. A significant number of the examined research are exploratory and utilize very small sample sizes, hence constraining statistical power and the generalizability. Diagnostic performance metrics are often unavailable, making it difficult to evaluate and compare biomarker efficacy in a clinically meaningful way.

The presence of significant clinicopathological variation among several genetic subgroups of familial FTD introduces further complexity. Biomarker expression may differ not only between genes but also among mutation carriers within the same family, and this heterogeneity is not generally acknowledged in the literature. Furthermore, the overlapping biomarker profiles among many neurodegenerative disorders, such as Alzheimer’s disease, amyotrophic lateral sclerosis, and atypical parkinsonian syndromes, may result in misinterpretation and diminish specificity.

A further limitation results from the prevalence of cross-sectional studies. In the absence of longitudinal data, evaluating the temporal dynamics of biomarker changes during the disease progression, particularly in the presymptomatic phase, is challenging. Variations in study design, inclusion criteria, comparison groups, and the utilization of diverse test platforms create further variability and confound inter-study comparisons.

Several biomarkers are not exclusive to familial FTD. Nfl and GFAP levels are elevated in various neurodegenerative diseases, including AD, to similar extents. Despite their variations across diseases, clinical cut-offs to differentiate between neurodegenerative diseases have not yet been established. The absence of specificity can result in diagnostic ambiguity, particularly in early or atypical cases.

In conclusion, fluid biomarkers present a significant potential for enhancing the diagnosis and monitoring of disease progression in familial FTD. However, their clinical application is currently constrained by challenges related to specificity, heterogeneity, sample availability, technological limitations, and economic viability. Addressing these limitations will require larger, multicenter, genotype-stratified longitudinal studies using harmonized protocols and standardized biomarker panels.

## 4 Conclusion

This review summarizes the recent fluid biomarker findings in familial frontotemporal dementia with respect to common genetic mutations. Several studies have been conducted in recent years on the fluid markers associated with familial FTD. In this review, we highlight fluid biomarkers in the blood and CSF that contribute to clinical diagnosis, disease progression surveillance, and pathophysiological mechanisms of familial FTD. Compared to expensive genetic tests, convenient and cost-effective fluid biomarkers are promising for use as screening tools and provide important information for disease prognosis. Future research should prioritize large-scale, multicenter longitudinal studies to validate candidate biomarkers across genetically stratified FTD cohorts. Moreover, integrating fluid biomarkers with advanced neuroimaging and multi-omics profiling could enhance diagnostic precision and therapeutic monitoring. The development of standardized, cost-effective, and scalable biomarker assays remains essential for clinical translation.
